# Novel anodic oxide film with self-sealing layer showing excellent corrosion resistance

**DOI:** 10.1038/s41598-017-01549-y

**Published:** 2017-05-02

**Authors:** Yinghao Wu, Wenjie Zhao, Wurong Wang, Liping Wang, Qunji Xue

**Affiliations:** 1 Key Laboratory of Marine Materials and Related Technologies, Zhejiang Key Laboratory of Marine Materials and Protective Technologies, Ningbo Institute of Materials Technology and Engineering, Chinese Academy of Sciences, Ningbo, 315201 China; 2 School of Materials and Engineering, Shanghai University, Shanghai, 200000 China

## Abstract

In the present work, the novel anodic oxide film (AOF) with self-sealing layer was successfully fabricated on 2024Al alloys by using an improved anodic oxidation method. The presence of the self-sealing layer on the porous layer of AOF was verified by Field emission scanning electron micro scope. Confocal laser scanning microscope (CLSM) and X-ray photoelectron spectroscopy (XPS) were used to evaluate the morphology and the corrosion products of the AOF after salt spray test. The microhardness test showed that the self-sealing AOFs still displayed high hardness even after salt spray test. Electrochemical test and salt spray test results illustrated the excellent corrosion performance of the novel structured self-sealing anodic oxide film (SAOF) compared with common porous AOFs. The narrow diameter makes it difficult for chlorine ions ingress into the pores of SAOFs. The self-sealing layer played an important role in protecting the SAOF from corrosion.

## Introduction

Aluminum and aluminum alloys are widely used in the aerospace, automotive industry and ocean environment due to their excellent performances, such as high specific strength, corrosion resistance and biocompatibility for both technical and economic considerations^1^. The 2024 aluminum alloys contain cooper, magnesium elements which were used to improve its mechanical properties. However, these elements in alloys appeared as active matrix, the microscopic galvanic couples formed by these elements make the alloy exceedingly sensitive to corrosion. To enhance the corrosion resistance and mechanical performance of aluminum alloys, the matrix was frequently treated by anodic oxidation^2^.

Among various techniques, anodic oxidation is a wide applied method to fabricate a passive oxide film on aluminum alloys^3^. In order to improve the corrosion resistance ability of anodic oxide film (AOF) on aluminum alloy, various electrolytes such as sulfuric acid^4–6^, chromic acid^7–9^, tartaric acid^10–12^, phosphoric acid^13–15^ and mixture of acid^16–19^ have been employed. The aluminum alloys cathode and the aluminum sulfate in the electrolyte hindered the dissolution of the AOFs formed by anodic oxidation, the pore size on the surface was reduced^20^. The oxalic acid added in the electrolyte can reduce the current density of the active region^21, 22^, and electric-field intensity of each cross-section of the oxide film tended to be identical. The addition of weak acid in electrolyte decreased the aggressiveness of the acid mixture, improved the formation of oxide layer and increased the thickness of films^23^. Therefore, a novel structured AOF with low surface roughness self-sealing layer showing excellent anti-corrosion performance was obtained successfully.

During the past several decades, lots of investigations on aluminum anodic oxidation were carried out to study the formation mechanism of AOFs^24, 25^. Generally, the typical AOFs contain a thin compact barrier layer and a thick porous layer, which is generally 10^3^–10^4^ times thicker than barrier layer. The film has increased the hardness, wear resistance and corrosion resistance of Al alloys. The influence of corrosion resistance of anodic oxide film responded to the film thickness^26^ and the morphology^27^. The element which influenced the growth of AOF such as current density^28^, temperature^29^, anodizing time^30^, composition^31^ and concentration^32^ of electrolyte will influence its corrosion behavior. Most previous research was devoted to study the influence factor on porous layer, such as pore size, pore morphology. The porous layer has been sealed by several kinds of anti-corrosion materials to increase the corrosion resistance of AOFs.

In the ocean industry, whenever anodized Al alloys are used in service, the layer must be either painted or sealed. Several works have evaluated several kinds of coatings to enhance the anti-corrosion performances of AA2024 AOF. Grégory Boisier *et al*.^33^ investigated four monocarboxylic acids with different carbon chain lengths as a post-treatment for sealing AA2024 AOFs. It was shown that the organic film formed very fast and contributed to the improvement of anti-corrosion of the sealed AOFs, the formation of aluminum soap owned hydrophobic performances to the surface and thus supply a protection of corrosion compared to untreated specimens.

Cerium sealing treatment was cooperated with investigate the practicability of *in-situ* TiB2p/A356 composite for anti-corrosion purpose by Moutarlier *et al*.^34^ Cerium oxide and cerium hydroxide were the main chemical composition for the sealing layer, which was composed of spherical deposits. The results indicated that the coordination effect of anodized film and cerium sealing layer resulted in a higher level of protection in a chloride-containing environment than a single anodized film for TiB2p/A356 composite, which was contributed to that the sealing layer completely sealed the pores and also hided most defects of anodic film.

V.R. Capelossi *et al*.^2^ anodized AA2024 Al in a tartaric-sulfuric acid bath and subsequently protected either by typical Cr-free water sealing treatment or by using of a hybrid sol-gel coating. The results illustrated that the treatment with the hybrid sol-gel strengthened the anti-corrosion properties of the pores compared to the common water sealing, deterring the access of aggressive species to the barrier layer.

Based on the above results, it was found that the sealed AOF have superior corrosion resistance when compared with unsealed AOF. However, the sealed layer on aluminum need a post-treatment, which was prone to wear, poor aging-resistance, got chalking and infected the appearance of the AOF. Until now, research on the *in-situ* growth of smooth outer surface of AOFs which contain self-sealing layer on the porous layer by an improved anodic oxidation was investigated little.

In this work, design and fabricate smooth and compact AOFs on aluminum was investigated. The self-sealing layer on the porous layer was fabricated via improved anodic oxide by adding oxalic acid and aluminum sulfate in electrolyte and replaced the graphite cathode by aluminum alloy. The novel self-sealing layer structure behaved excellent corrosion resistance, especially the corrosion protective in salt spray test (SST). Surface morphology analysis and electrochemical test were used in this work to investigate the formation and corrosion behavior of the novel structured AOFs and the anticorrosion mechanism of the AOFs.

## Results and Discussion

**Figure 1:**
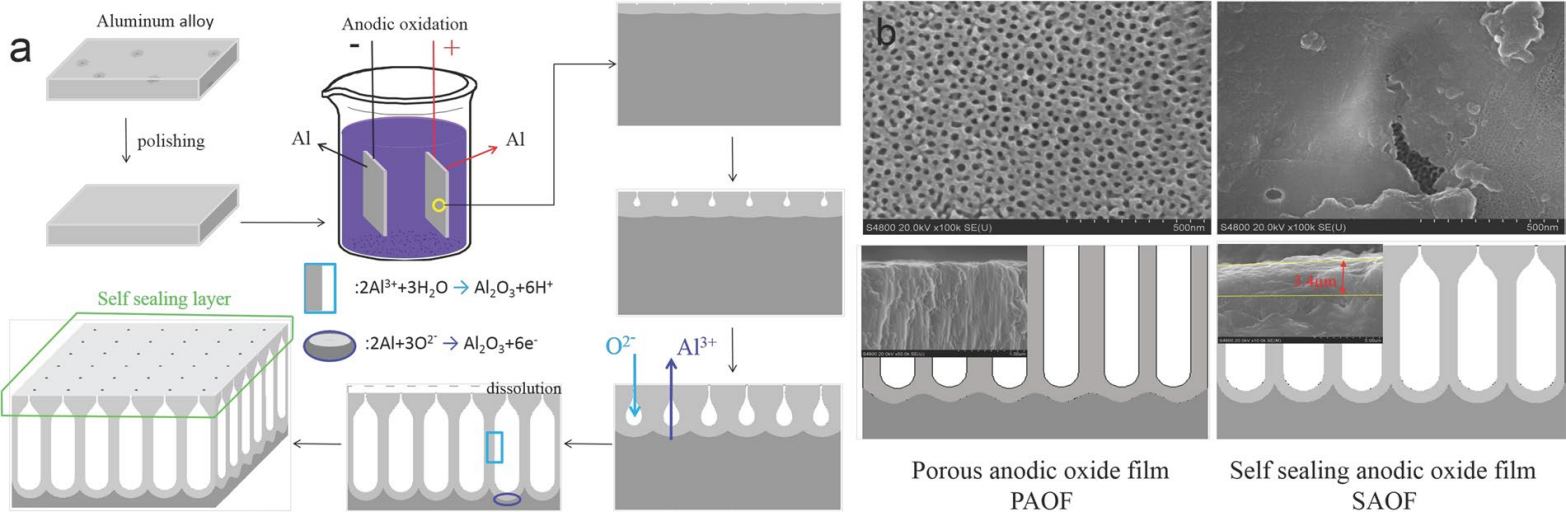
The formation of self-sealing layers on AOF in anodic oxidation and the morphology of PAOF and SAOF.

The AOFs were prepared by using the common anodizing method. The electrochemical reactions^35^ at pore wall and barrier layer/Al substrate interface were shown in Fig. 1. The formation of the self-sealing layer on AOFs was attributed to the adding of oxalic acid and aluminum sulfate in electrolyte and aluminum cathode. The aluminum alloys cathode and the aluminum sulfate in the electrolyte increased the Al^3+^ concentration which hindered the dissolution of the outer surface of AOFs^20^. The oxalic acid added in the electrolyte reduced the current density of the active region^21, 22^, and the electric-field intensity of each cross-section of the oxide film tended to be identical. The addition of weak acid in electrolyte decreased the aggressiveness of the acid mixture and increased the thickness of AOFs^36^. The self-sealing layer was named for comparing with the typical PAOFs shown in Fig. 1b. The porous layer can be found below the self-sealing layer, the diameter of the pores for porous layer and the total thickness of two types of AOFs was about the same size (30–50 µm). The diameter of pores for PAOFs was in a range of 30–50 nm. For SAOFs, the surface layer was compact and nano-scaled pores presented on the outer surface of SAOFs in Fig.1b which was hardly seen by our eyes. The pore diameter of porous layer below the surface layer were about 20–35 nm shown in Fig. 1b at the defect region. The thickness of the self-sealing layer was about 3–4 µm. The pores’ outlet on the interface of electrolyte and sample surface should beyond a critical pore size and wide enough to make sure the electrolyte penetrated into the pores and the Al^3+^ could translate from the pores into electrolyte^37^.

In corrosion process, the chloride ions and other corrosive medium competitively adsorbed on the surface of AOFs and ingressed into the pores which lead the corrosion of AOFs^38^. In Fig. 1b, the corrosion medium such as chloride ions could easily ingressed into the pores of PAOF. In The narrow outlet of the pores of SAOFs lead to the chloride ions ingressed into pores of AOFs difficultly.

**Figure 2:**
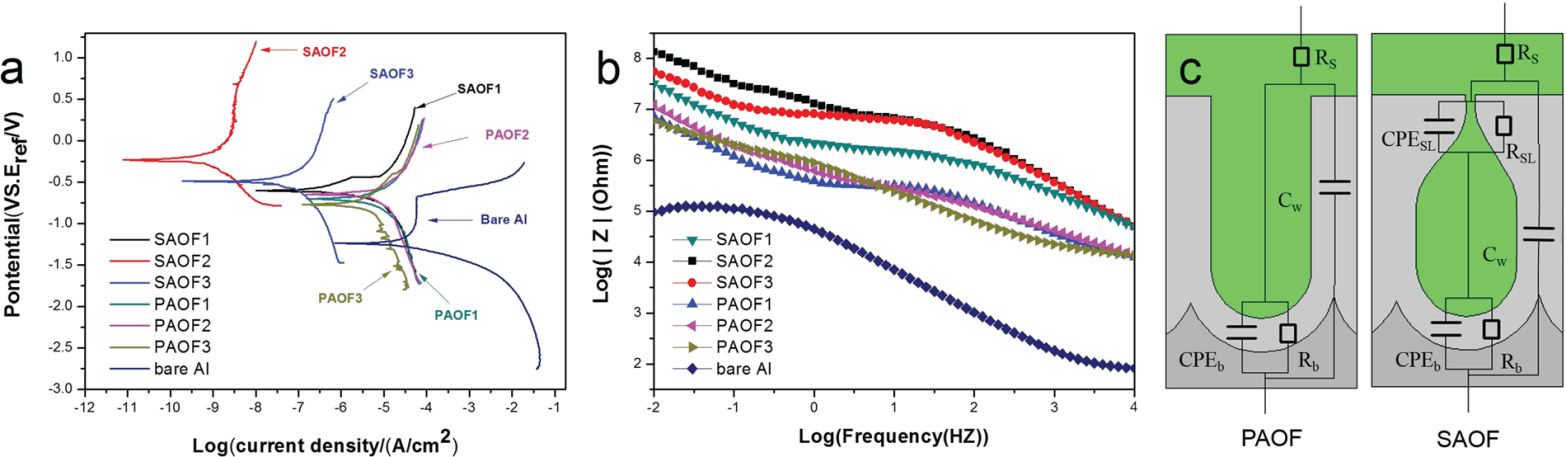
Potentiodynamic polarization, bode plot and equivalent circuits of self-sealing AOFs, PAOFs and Al alloy.

Potentiodynamic polarization was carried out for the SAOFs and PAOFs in a 3.5% NaCl solution, the obtained results were given in Fig. 2. The self corrosion current density (i_corr_) values received for self-sealing AOFs are one (SAOF1) to three (SAOF2) orders of magnitude lower than PAOFs respectively. From Fig. 2a, it shown that in the anodic branch exist a step rise in current density above the self corrosion potential (E_corr_) indicating the pit corrosion appeared on the bare Al alloy. However, the current density of AOFs higher than E_corr_ changed gently in anodic region illustrating the passive nature of the anodized Al alloys. For the two types of AOFs, they displayed higher E_corr_ and lower i_corr_ than Al alloy, which indicated the two types of AOFs increased the corrosion resistance of Al alloys. In the case of self-sealing AOFs, the self-sealing layer of SAOF hindered the chlorine ions ingress into the pores of films, the higher E_corr_ and lower i_corr_ indicating better corrosion resistance.

The electrochemical impedance spectra (EIS) of the SAOFs, PAOFs and Al alloy have been investigated in this study. Figure 2b showed the impedance spectra (Bode plots) of SAOFs, PAOFs and bare Al alloy. The value of impedance at low frequency (10 mv) is an intuitive parameter that can be used to compare the corrosion resistance of two types AOFs. The impedance values of SAOFs were higher than 10^8^ Ωcm^2^, which were about one order of magnitude larger than the PAOFs and three orders of magnitude larger than Al alloy. The high impedance modulus of SAOFs was attributed to the self-sealing layer by prevent chlorine ions from ingressing into the porous layer though the nano-scaled outlet of pores.

**Figure 3:**
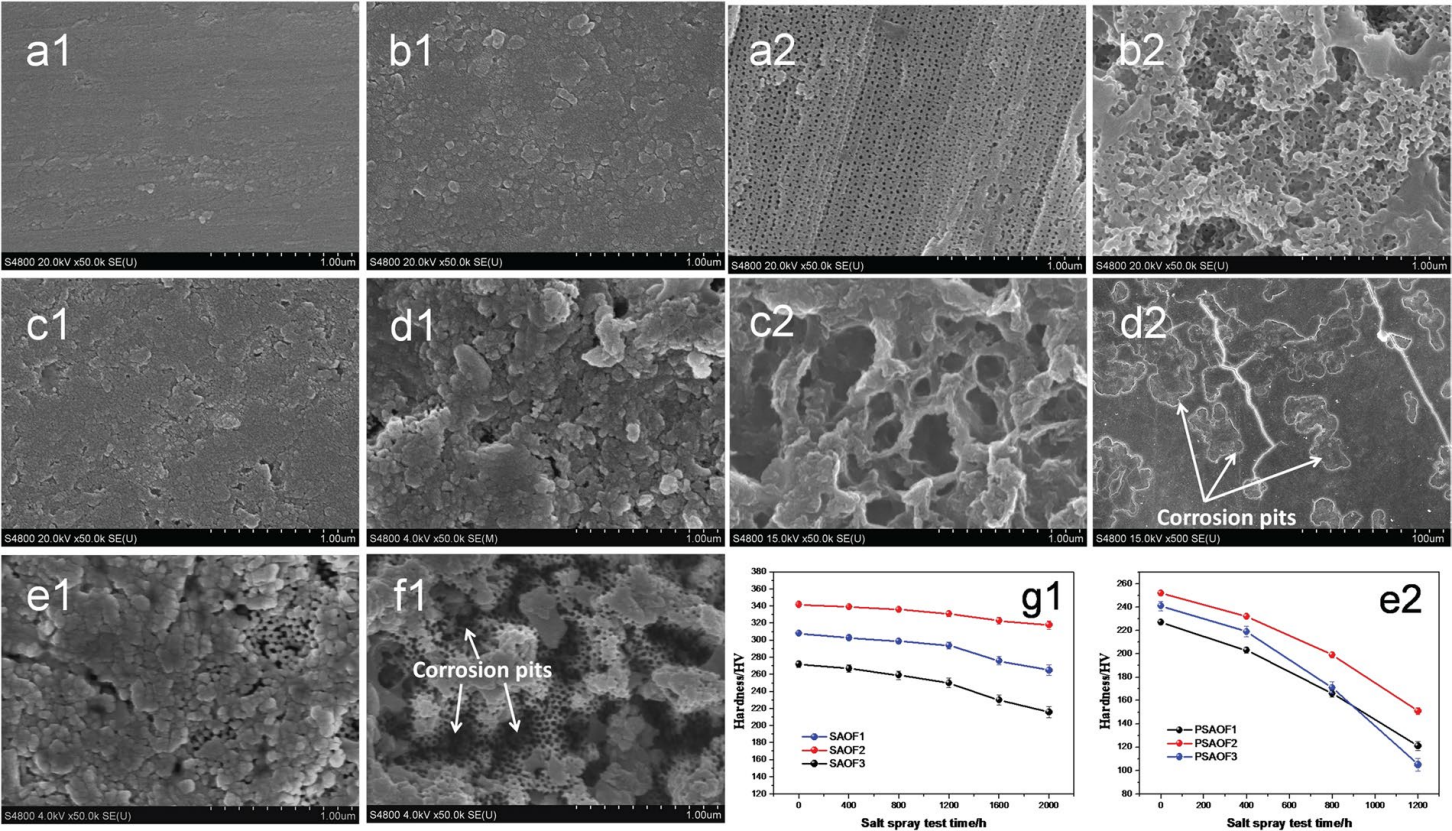
Surface morphologies and microhardness of SAOF and PAOF corroded with different SST time, (a1 and a2) 0 h, (b1 and b2) 400 h, (ca and c2) 800 h, (d1 and d2) 1200 h, (e1) 1600 h, (f1) 2000 h, g1 and e2 showed microhardness decreased with time.

The equivalent circuits shown in Fig. 3c were obtained by curve fitting analysis of SAOFs and PAOFs. For PAOFs, the corrosion resistance was characterized by the resistance R_b_ and capacitance CPE_b_. The R_b_ and CPE_b_ described the barrier layer, for it is fairly homogeneous and free of defects, it was the main corrosion resistance of the PAOFs^39^. R_S_ responded to the resistance of electrolyte which was full of pores. For SAOFs in Fig. 2c, the capacitive response of the self-sealing layer was represented by a pure capacitor with a n value^40^ which was 0.95, 0.89 and 0.83 for SAOF2, SAOF3 and SAOF1 respectively indicating that CPE_SL_ was more capacitive in nature. The parameter n is the frequency dispersion factor and can be considered as a capacitor when it reached 1. The CPE_SL_ for SAOFs associated to the changes of EIS response according to the formation of self-sealing layer out of the porous layer compared with PAOFs. The excellent corrosion resistance associated to the R_SL_ represented the important role of self-sealing layer on hindering the chlorine ions ingressed into the porous layer of AOFs. Therefore, for the self-sealing sample the resistive and CPE elements characterizing the EIS response around the pores of the AOFs were denominated CPE_SL_ and R_SL_ to differentiate from the common PAOFs. The C_W_ was responded to the capacitive behavior of the pore wall. The capacitive behavior of pore wall represented a constant phase element rather than a pure capacitor, the value changed with the thickness of porous layer. The electrical equivalent circuit of SAOF was similar to the previous research for PAOF sealed with corrosion resistance materials, which verified the anti-corrosion role of self-sealing layer played on the common PAOF surface^2, 33^.

The surface morphologies of SAOFs corroded with different salt spray tests (SSTs) time were shown in Fig. 3. Before the SST (Fig. 3a1), the surface of SAOFs was smooth and compact, the pores existed on the outer surface were nano-scaled which can’t be distinguished extremely. In Fig. 3e1 and f1, it can be found that there existed uniform pores under the self-sealing layer, the corroded surface morphology verified the novel multilayer structure of SAOFs which shown in Fig. 1b. In SST process, chlorine ion adsorbed on the surface and ingressed into the porous layer of AOFs which resulting in the corrosion of AOFs and was the key factor for the onset of passive film dissolution. The dissolution on surface destroyed the self-sealing layer and exposed the porous layer out. For the early 1600 h, the corrosion on SAOFs was light and slow. After that, the hollows on surface appeared and grew bigger and deeper. For 1600 h in Fig. 3e1, the porous layer exposed and the thickness of self-sealing layer remained to be several nanometers. No corrosion pit appeared on the surface, the uniform corrosion was the key corrosion style. However, for Fig. 3f1 (2000 h), the small sized (0.5–1 μm) corrosion pits spread all over the sample surface. Take the reverse into consideration, for Fig. 3d1, the nano-scaled hollows can be regarded as the germination of pitting corrosion. The chlorine ions were easily absorbed on pores outlets of PAOF and ingressed into the pores. The chlorine ions absorbed on the surface and permeated into the Al substrate for SAOFs were much difficult than that of PAOFs, the dissolution on SAOF surface was slight. In Fig. 3d2, the dissolution on PAOF was serious and uncontrollable, the corrosion pits on PAOFs formed and grew quickly (50–200 μm). The cracks initiated and propagated on PAOF surface. By comparing with SAOFs and PAOFs, when the SST time was 1200 h, the corrosion pits on SAOF were about 200–600 nm, which illustrated that the corrosion resistance of SAOF was much better than PAOF.

Figure 3g1 showed the surface microhardness of SAOFs changed with SST time. The SAOF2 was selected to investigate the change of microhardness compared with the PAOF2. SAOF2 and PAOF2 were selected because of their high hardness and better corrosion resistance in the microhardness test and electrochemical test before salt spray test, and they represented the two kinds of typical microstructure of AOFs. Before salt spray test, the microhardness of SAOF was about 4–7 times higher than untreated Al alloys (56.5 HV). In Fig. 3g1, the microhardness decreased with SST time. In 0–1200 h, the hardness decreased slowly because the corrosion medium were slowly ingressed into the AOFs, and the hardness of self-sealing AOF decreased more slowly compared with the porous AOF which was due to the better corrosion resistance. As time goes on, the corrosion on surface become more serious and the microhardness of SAOF decreased fast. After 1200 h, the corrosive medium ingressed into the self-sealing layer of AOF, the corrosion on the SAOF surface got more serious and the sample surface dissolved quickly which lead to the decreased of surface microhardness. As the corrosion on SAOF surface getting complicated with the increase of SST time, the decrease of hardness could not be ignored. However, the SAOF2 still kept a high microhardness value even after been corroded in salt spray test for 2000 h which demonstrated SAOFs obtained in this work showing promising application in various fields. Compared with the SAOFs, the microhardness of PAOFs decreased fast in the early 1200 h (Fig. 3e2). For 1200 h, the hardness of PAOFs decreased to half of the value before SST. The chlorine ions absorbed on the surface and corroded the films, the dissolution on surface and corrosion pits caused the hardness decreased quickly.

**Figure 4:**
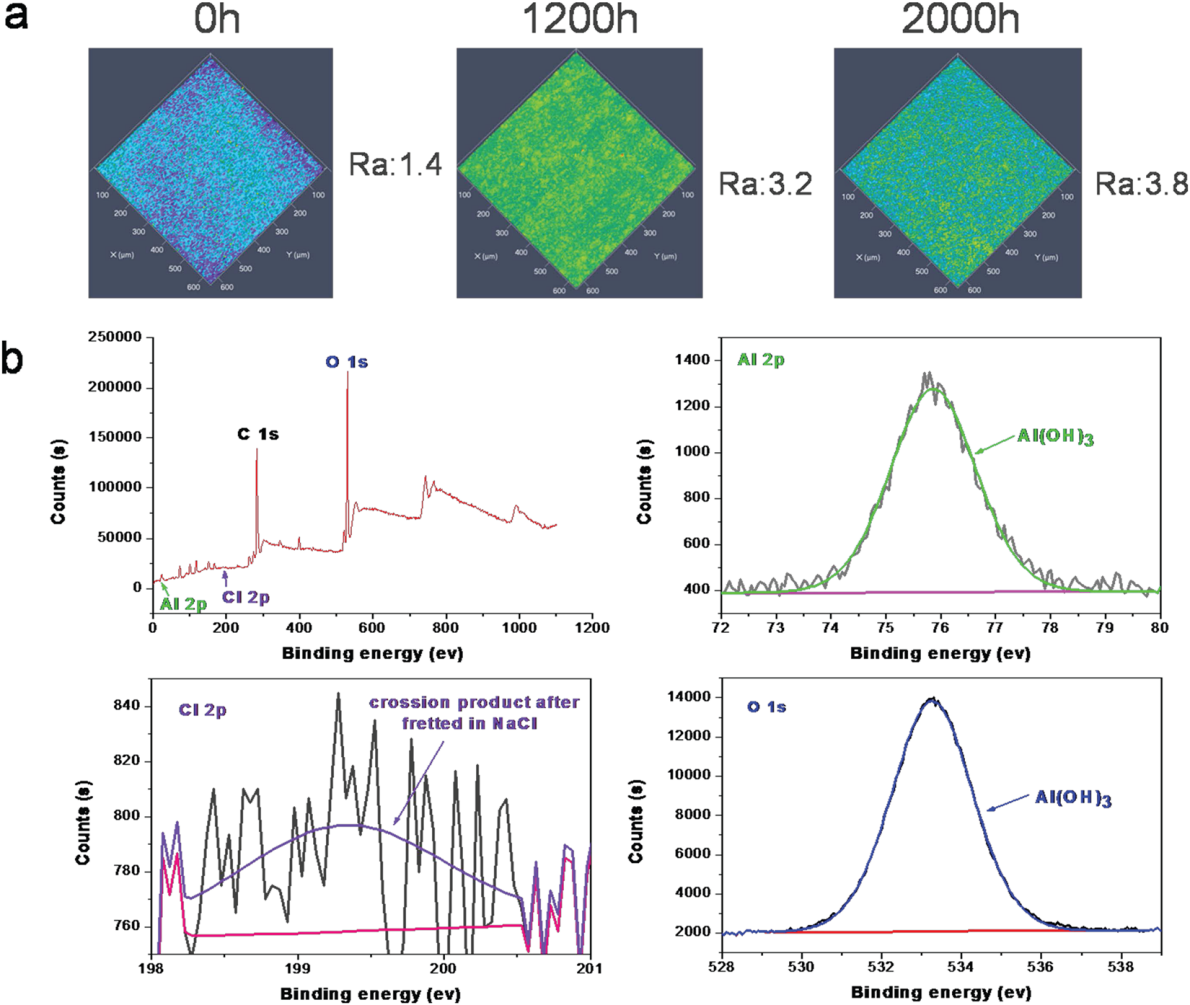
Surface roughness of SAOFs changed with salt spray test time and XPS analysis of corrosion product after salt spray test.

The 3D surface morphologies and surface roughness (R_a_) of SAOFs changed with SST time were shown in Fig. 4a. Before SST, the surface of AOFs was smooth and compact which represented low surface roughness. As time goes by, surface dissolution appeared on AOFs caused of chlorines ions adsorbed on surface and ingressed into the pores. The dissolution of film in salt spray resulted in surface roughness increased rapidly after 1200 h. The color changed on samples surface for 1200 h illustrated the surface were not smooth, there existed pits and protrusion on surface which were due to corrosion. For 2000 h, the sample AOF2 was less colorful indicating that the dissolution of film surface was slightly. The chlorine ions ingressed into the pores of SAOFs difficultly and the AOF2 represented the best corrosion resistance. Compared with the decreased surface roughness of PAOF, the SAOFs presented less change of R_a_ value which displayed the excellent corrosion resistance of self-sealing layer.

The de-convoluted XPS core level Al 2p, O 1 s and Cl 1 s spectra of SAOF2 was shown in Fig. 4b. It is evident that the broad spectra of Al 2p and O 1 s corresponded to the Aluminum hydroxide. De-convolution of the obtained spectrum indicated the presence of the peak of 2p3/2 at 75.85 eV corresponding to Al(OH)_3_. From the core level of O 1 s spectrum, the relative intensities of de-convoluted O component peaks the presence of metal hydroxides, respectively. The core level Al 2p spectrum of self-sealing AOF also showed the presence of aluminum hydroxides, respectively. In the present investigation, the presence of Al(OH)_3_ illustrated the hydration of Al_2_O_3_ and formation of Al(OH)_3_ during the salt spray test. The binding energy of Cl 1 s was approximately 199.3 eV, corresponding to corrosion products after fretted in NaCl, and it was believed that Al(OH)Cl_X_ was produced in SST after the sample immersed in salt fog for long time^41^. The obtained at.% of chlorine on SAOF and PAOF were about 4% and 23%, respectively. The results illustrated that the PAOF was corroded more serious. Therefore, based on the above results the corrosion reactions during salt spray test are given below:

$${{\rm{Al}}}_{2}{{\rm{O}}}_{3}+3{{\rm{H}}}_{2}{\rm{O}}\to 2{\rm{Al}}{({\rm{OH}})}_{3}$$

$${\rm{Al}}{({\rm{OH}})}_{3}+{{\rm{Cl}}}^{-}\to {\rm{Al}}({\rm{OH}}){{\rm{Cl}}}_{{\rm{X}}}$$

**Figure 5:**
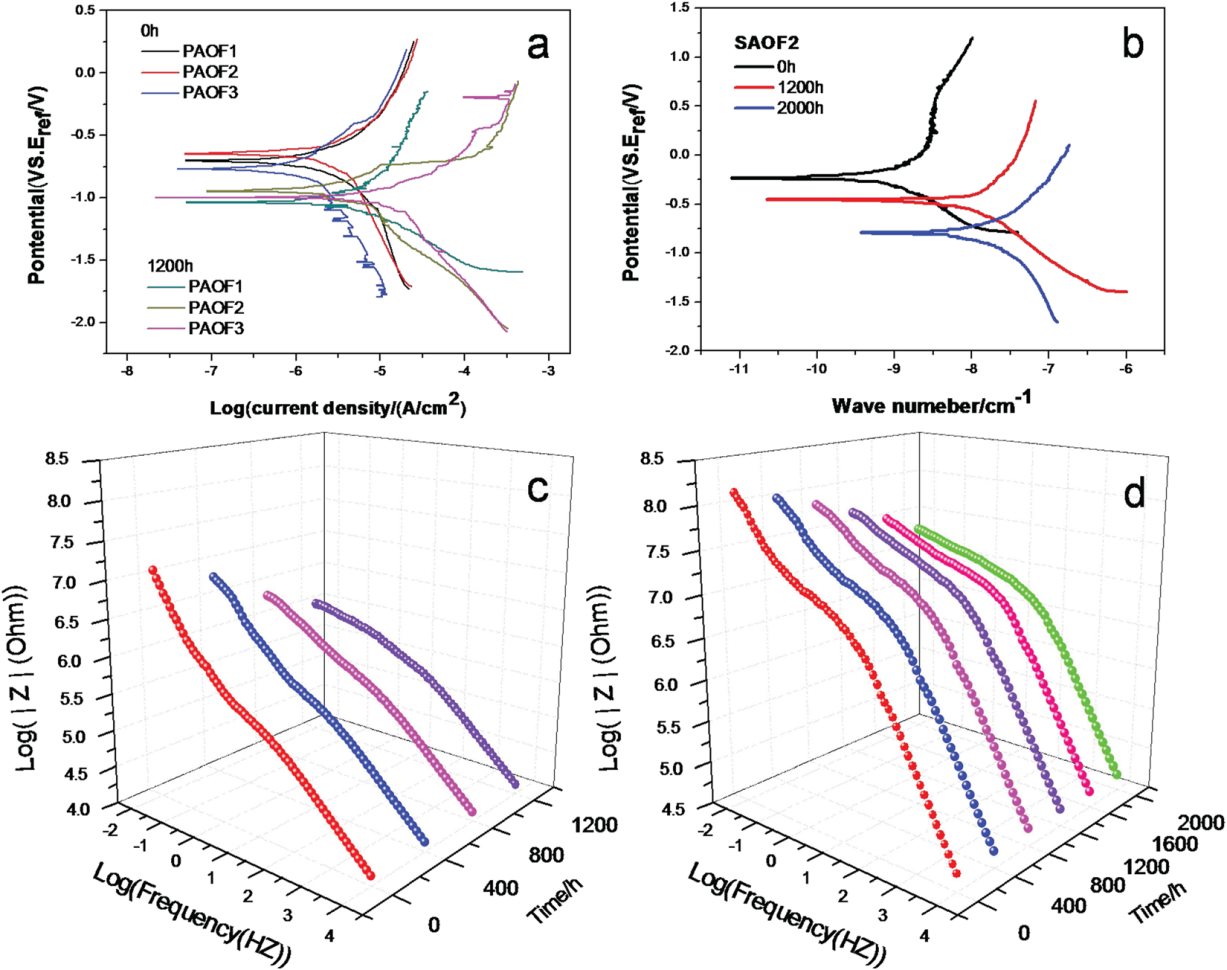
Potentiodynamic polarization, (**a**) (SAOF), (**b**) (PAOF) and impedance modulus of samples changed with SST time, (**c**) (SAOF), (**d**) (PAOF).

The chlorine ion selective absorbed ability of AOFs makes the dissolution on surface morphology was differently after SST. The corrosion resistance of SAOFs and PAOFs was decreased with the increase of SST time. After 1200 h SST, the PAOFs corroded extremely seriously. However, the SAOFs corroded for 2000 h in SST begin to form pits (Fig. 3f1). After the exposure of porous layer of SAOFs, the chlorine ions ingressed into the porous layer became easier and the corrosion on the films quickly and the anti-corrosion property of SAOFs decreased. In Fig. 5b, the Potentiodynamic polarization changed with SST time illustrated the SAOFs showed a better corrosion resistance than PAOFs. For the early 1200 h SST for SAOFs, the self corrosion potential decreased and the self corrosion current density increased slowly due to the barrier effect of self-sealing layer. Though corroded in salt fog for 1200 h, self corrosion potential and the self corrosion current density estimated the SAOF were still displayed a better corrosion resistance than PAOFs. After 1200 h, the self-sealing layer was corroded and the porous layer exposed, the self corrosion potential decreased and the self corrosion current density increased quickly. In Fig. 5a, the self corrosion potential decreased and the self corrosion current density increased fast, which illustrated the corrosion resistance of PAOFs decreased quickly just after 1200 h SST.

Figure 5d showed the impedance spectra of SAOF2 changed with SST time. It can be found that the plateau for SAOF2 at the middle range of frequency existed stably before 2000 h SST, which could be attributed to the strengthened corrosion resistance. With times goes by, the plateau for PAOF disappeared after 400 h SST and 1600 h SST for SAOF which meant serious corrosion appeared on the AOFs, the disappearance of plateau was owing to the high Permeability of the chloride ions through the porous layer with the SST time^42^. Compared with SAOF and PAOF, the novel structure of surface layer made the Permeability of the chloride ions become difficult. When the porous layer exposed due to the dissolution of self-sealing layer, the high mobility of the chloride ions ingressed into the porous layer decreased the corrosion resistance of AOFs. The value of impedance was decreased with time faster and faster. Compared with the PAOFs, the impedance of SAOFs decreased much slowly. For PAOFs after SST in Fig. 5c, the chlorine ions absorbed on the surface quickly and ingressed into the pores easier than SAOFs, which caused the dissolution of surface seriously, the size of pits grew fast and the thickness of AOF decreased quickly. The chlorine ions ingressed into the pores and permeate into substrate through the barrier layer.

**Figure 6:**
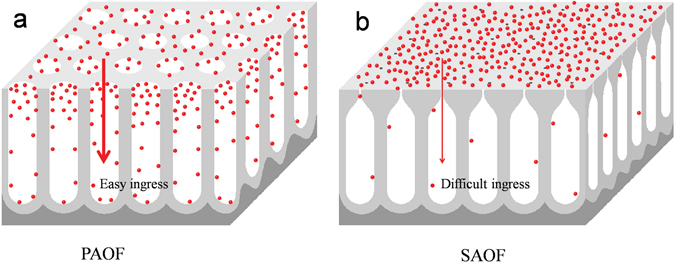
The mechnism of the anti-corrosion of SAOF and PAOF.

In Fig. 6a, the chloride ions could easily ingressed into the pores of PAOF and absorbed on the pore walls. In Fig. 6b, the narrow outlet of the pores of SAOFs lead to the chloride ions ingressed into pores of AOFs difficultly. The chlorine ions migration occurred once a critical anodic potential or critical adsorbed chloride ions concentration was reached. The chloride ions migrated from the liquid drop/AOFs interface into the passive AOFs, resulted in initiation of pitting corrosion^43^. With time goes by, the ingress of chlorine ions caused the surface dissolution and the pitting corrosion showed in Fig. 3. The concentration of chloride ions adsorbed on AOFs surface increased with time. The dissolution of surface make the corroded surface were easier be attacked by chlorine ions. After that, the microhardness decreased (Fig. 3), the dissolution of AOFs surface in salt fog accelerated (Fig. 4a) and the self corrosion potential decreased (Fig. 5).

A novel structured SAOF characterized with self-sealing layer was fabricated by improved anodic oxidation, the composition and concentration of electrolyte, current density and the aluminum cathode was tailored to form the self-sealed AOFs. The electrochemical test and salt spray test were carried out to investigate the corrosion performance of SAOFs and PAOFs. The CLSM and XPS were used to study the surface morphology and corrosion products after SST. The mircohardness test illustrated the hardness of SAOFs decreased slowly with SST time. SEM images and 3D morphology of surface estimated the SAOFs appeared fast corrosion after 1600 h SST. The electrochemical test, SEM image and 3D morphology illustrated the self-sealing layer play a more important role in corrosion preventive compared with porous layer of AOFs. The narrow outlets of the pores on SAOFs successfully prevent chlorine ions from ingressing into the pores of SAOFs. The corrosion pits appeared on the surface of SAOFs until the SST was 2000 hours. The corrosion resistance of SAOF after been corroded for 1200 h in SST was still better than PAOF without SST. The results in this work provided a new train of thought for protecting aluminum alloys from corrosion.

## Method

The 2024 Al substrates were ground by emery paper (no. 800, 1200, 1500, 2000) gradually, and mechanically polished until polish scratch can’t be seen by eyes. Then the substrates were ultrasonically cleaned in acetone and ethanol for 10 min each. The anodic oxidation process was carried out using constant current operation in 45–120 mg/mL sulfuric acid, 10 mg/mL oxalic acid and 5 mg/mL aluminum sulfate solution for 30 min, the anodic oxidation current density was tailored from 3.5 A•dm^−2^ to 6.5 A•dm^−2^ (included 3.5, 5 and 6.5). In order to enhance the growth speed and alleviate the dissolution speed of oxide film, the Al alloy was used as both of anode and cathode in this work. After being ultrasonically cleaned with deionized water, the obtained specimens were dried at 60 °C for 10 min. The sample anodized in 45 mg/mL, 90 mg/mL and 180 mg/mL sulfuric acid bath with the current density of 5 A•dm^−2^ was marked as AOF1, AOF2 and AOF3, respectively.

Surfaces structures of self-sealing anodic oxide films (SAOFs) and porous anodic oxide films (PAOFs) fabricated in this work were studied by a Confocal laser scanning microscope (CLSM) and a field emission scanning electron microscope (SEM, FEI Quanta 250 FEG, USA) under a vacuum environment, with an accelerating voltage of 20 KV. The samples were also investigated using a PHI-5702 multi-functional X-ray photoelectron spectroscope (XPS, Perkin-Elmer, USA) to characterize the corrosion products after SST, using monochromatic Al Ka irradiation and the chamber pressure was about 3 ×10^−8^ Torr. The binding energy of adventitious carbon was provided as a base reference. The micro hardness was measured by a standardized Vickers hardness test device using 300 g load and the dwell time was 10 s on the surface of AOFs. The microhardness value presented in this work is an average of at least 10 measurements.

Electrochemical corrosion tests were investigated by an electrochemistry workstation (Modulab, Solartron, USA) using potentiodynamic polarization in a three-electrode system: the specimen was treated as the working electrode with a evaluate area of 1 cm^2^, a platinum wire counter electrode, and a saturated calomel reference electrode. Electrochemical measurements were implemented in 3.5 wt.% NaCl aqueous solution at room temperature. Tafel polarization curves were dynamically performed on respect to the reference at a scanning rate of 2 mV/s. The electrochemical impedance spectroscopic measurements were executed in the frequency range from 0.01 Hz to 10^3^ Hz.

The corrosion performance of the novel structured SAOFs and PAOFs were investigated using corrosion accelerated experiments (Q-FOG, Q-lab Corporation, USA), according to Standard Method ASTM B117^44^ in 5 wt% NaCl solution (6.5 < pH < 7.2) at (35 ± 2) °C with a spray flow rate of 40 mL/h. The samples were periodically taken out from the chamber for visual evaluation of the extent of corrosion. The corrosion progress was evaluated from micrographs of the exposed surfaces that were recorded with increasing exposure times. The samples were taken out periodically to test the roughness, 3D morphologies, microhardness and electrochemical properties of the samples when the corrosion time was 400 h, 800 h, 1200 h, 1600 h and 2000 h. The samples were washed with flowing water at 35 °C to remove the saline deposits on the surface before electrochemical test. Then, the samples were dried at room temperature for 20 min.
